# The global burden of sickle cell disease in children under five years of age: a systematic review and meta-analysis

**DOI:** 10.7189/jogh.08.021103

**Published:** 2018-12

**Authors:** Elizabeth Wastnedge, Donald Waters, Smruti Patel, Kathleen Morrison, Mei Yi Goh, Davies Adeloye, Igor Rudan

**Affiliations:** Centre for Global Health Research, The Usher Institute for Population Health Sciences and Informatics, University of Edinburgh, Scotland, UK

## Abstract

**Background:**

Sickle cell disease (SCD) is a common haematological disorder, affecting millions of people worldwide. It is most prevalent in malarial endemic areas in the tropics where outcomes are often poor due to resource constraints, resulting in most children dying before reaching adulthood. As increasing progress is made towards reducing under 5 mortality from infectious causes, non-communicable diseases (NCDs) including SCD have risen to the forefront of the global health agenda. Despite this, the global mortality burden of SCD remains poorly understood. This study aimed to estimate the incidence and mortality of SCD in children under 5 years of age in order to inform policy and develop sustainable strategies to improve outcomes.

**Methodology:**

We performed a systematic literature search of Medline, EMBASE, Journals@Ovid, and Web of Science for studies on the incidence and mortality of SCD in children under 5, with search dates set from January 1980 and July 2017. We conducted random effects meta-analysis to obtain pooled meta-estimates of birth prevalence and mortality rates globally, and for each World Health Organization (WHO) region.

**Results:**

67 papers were found with relevant data. 52 contained data on incidence and prevalence and 15 contained data on mortality. The overall pooled estimate of mortality from the limited data available was 0.64 per 100 years of child observation (95% CI = 0.28-1.00) with the highest rate seen in Africa 7.3 (95% CI = 4.03-10.57). The global meta-estimate for the birth prevalence of homozygous sickle cell disease was 112 per 100 000 live births (95% CI = 101-123) with a birth prevalence in Africa of 1125 per 100 000 (95% CI = 680.43-1570.54) compared with 43.12 per 100 000 (95% CI = 30.31-55.92) in Europe.

**Conclusion:**

There were a number of limitations in the depth and breadth of available data however it is clear that both the highest prevalence and highest mortality of SCD is in Africa. In order to address this burden, there is a need for national comprehensive newborn screening to identify patients, and the development of holistic SCD care programmes to provide therapeutics and education for families and children with SCD. This targeted funding should form part of a broader increased global focus on NCDs in childhood.

Sickle cell disease (SCD) is the most common genetic haematological disorder, accounting for over 305000 births in 2010, with millions of people currently affected across the globe [1,2]. Sickle haemoglobin (HbS) is a variant of normal adult haemoglobin, caused by a mutation in the HBB gene and inherited as an autosomal recessive Mendelian trait [2]. Red blood cells (erythrocytes) with HbS become deformed under stress, forming a classic ‘sickle’ shape [3]. Although heterozygotes (‘sickle cell trait’) are usually asymptomatic, patients who have inherited HbS alleles from both parents suffer from Sickle Cell Anaemia (SCA), the most common and severe form of SCD (a term that technically refers to any condition in which the production of HbS causes symptomatology, and can result from a broad range of inherited HBB mutations) [3]. In patients with SCD, sickling of erythrocytes causes haemolysis, reduces the oxygen carrying capacity of erythrocytes, and can result in episodic microvascular occlusion leading to tissue ischaemia and painful ‘crises’ with serious and often life-threatening consequences [3].

The global distribution of sickle cell disease is closely linked to the natural protection against malaria afforded to individuals who are heterozygous for the sickle cell mutation. This selective advantage throughout human history has resulted in the distribution of HbS mutations closely reflecting the global malaria incidence, focused around the tropics [4]. Unfortunately, many tropical countries do not have the necessary resources required to provide the complex care required for SCD patients, and resulting outcomes are typically poor. While in high-income settings the current life expectancy for patients with SCA is estimated to be between 45-55 years of age, in low- and middle-income countries (LMICs) it is thought that most children die before reaching adulthood, with more than 500 children with SCD dying every day because of poor access to appropriate treatment [2,5,6]. This stark disparity is emphasised by current estimates suggesting 90% of SCD occurs in LMICs, and 90% of children with SCD in LMICs die before their 5th birthday [6].

As increasing progress is made towards reducing under 5 mortality from infectious causes, non-communicable diseases (NCD) have risen to the forefront of the global health agenda. SCD is recognised as a significant cause of NCD-related childhood mortality and has been identified as an area requiring specific focus in order to meet the sustainable development goals [1,6]. Despite this, the global burden of SCD remains poorly characterised. This study aims to estimate the incidence and mortality of SCD in children under 5 years of age so as to better inform policy and develop sustainable strategies to improve outcomes.

## METHODS

### Search strategy and selection criteria

We conducted a systematic review of the literature in accordance with the PRISMA guidelines [7]. The search was conducted using the following databases: Medline, EMBASE, Journals@Ovid and Web of Science. The search terms used are detailed in **Table 1**. We further screened the reference lists of relevant papers and review papers for eligible articles. Inclusion and exclusion criteria are detailed in **Table 2**. Searches were limited to between January 1980 and July 2017, and there were no publication status or language restrictions applied. The search was performed by two independent reviewers to minimise bias.

**Table 1:** Search terms*

**1.**	exp mortality/ or exp morbidity/ or exp prevalence/ or exp incidence/
**2.**	(inciden* or prevalen* or mortality or morbidity or “case fatality”).ti,ab.
**3.**	(burden adj2 disease*).ti,ab.
**4.**	exp epidemiologic methods/ or exp data collection/ or exp health surveys/ or exp morbidity/ or exp incidence/ or exp prevalence/ or exp mortality/ or exp disease notification/ or exp epidemiological monitoring/
**5.**	1 or 2 or 3 or 4
**6.**	exp An?emia, Sickle Cell/
**7.**	(sickle cell adj2 (disease* or an?emi* or disorder)).ti,ab.
**8.**	sickle h?emoglobin.ti,ab.
**9.**	splenic sequestration.ti,ab.
**10.**	6 or 7 or 8 or 9
**11.**	5 and 10
**12.**	limit 11 to (human and child)
**13.**	limit 12 to yr = ”2002 - 2017”
**14.**	remove duplicates from 13
**15.**	limit 12 to yr = ”1980 - 2001”
**16.**	remove duplicates from 15
**17.**	14 or 16

*This applies to Medline, EMBASE and Journals@Ovid.

**Table 2:** Inclusion and exclusion criteria

**Inclusion criteria:**
Contained data for children <5 years old
Diagnosis of sickle cell disease including homozygous sickle cell disease and heterozygous HbSC or HbS-βth
Reported on mortality, incidence or prevalence of sickle cell disease
Published between January 1980 – July 2017
Prospective or retrospective cohort studies or cross-sectional studies
**Exclusion criteria:**
Other minor heterogenous phenotypes of sickle cell disorder eg, HbSD Punjab or HbSO Arab
Unclear methodology
No population denominator
Phenotypic study
Results not stratified by age

HbSC – hemoglobin S and C, HbSβ – hemoglobin S plus B thalassemia, HbSD – hemoglobin S and D, HbSO – hemoglobin S and O

### Data extraction

Data were extracted on study duration, location, WHO region, sample size and birth prevalence, and mortality from SCD. Where studies had overlap in data, the most recent study was used. Data extraction was also performed by two independent reviewers to minimise bias.

### Definitions

Homozygous sickle cell disease was defined as being homozygous for HbSS or heterozygous with HbS-βth or HbSC; heterozygous sickle cell trait was defined as having the genotype HbAS. Genotype was ascertained by high performance liquid chromatography or iso-electric focussing.

### Quality assessment

For each full text selected, we checked for sampling (was it representative of a subnational or national population?), statistical analysis (was it appropriate for the prevalence or mortality estimate?), and case ascertainment (was it based on standard screening tests, informal interviews, or not reported?). Studies were graded as high (4-5), moderate (2-3), or low quality (0-1). All low quality studies were excluded from the review.

### Statistical analysis

A random effects meta-analysis was performed on the extracted crude data to obtain pooled estimates of mortality and of birth prevalence for both homozygotes and heterozygotes for sickle cell. When estimating mortality, where studies did not provide mortality rate or child years of observation, child years of follow up was estimated by multiplying the sample population by the duration of the study. Global meta-estimates were obtained as well as meta-estimates for each world region as defined by the WHO. Due to limited regional data, we did not conduct sub-group (sensitivity) analysis based on the assessment of study quality. All statistical analyses were conducted on STATA (Stata Corp V.13, Texas, USA).

## RESULTS

**Figure 1:**
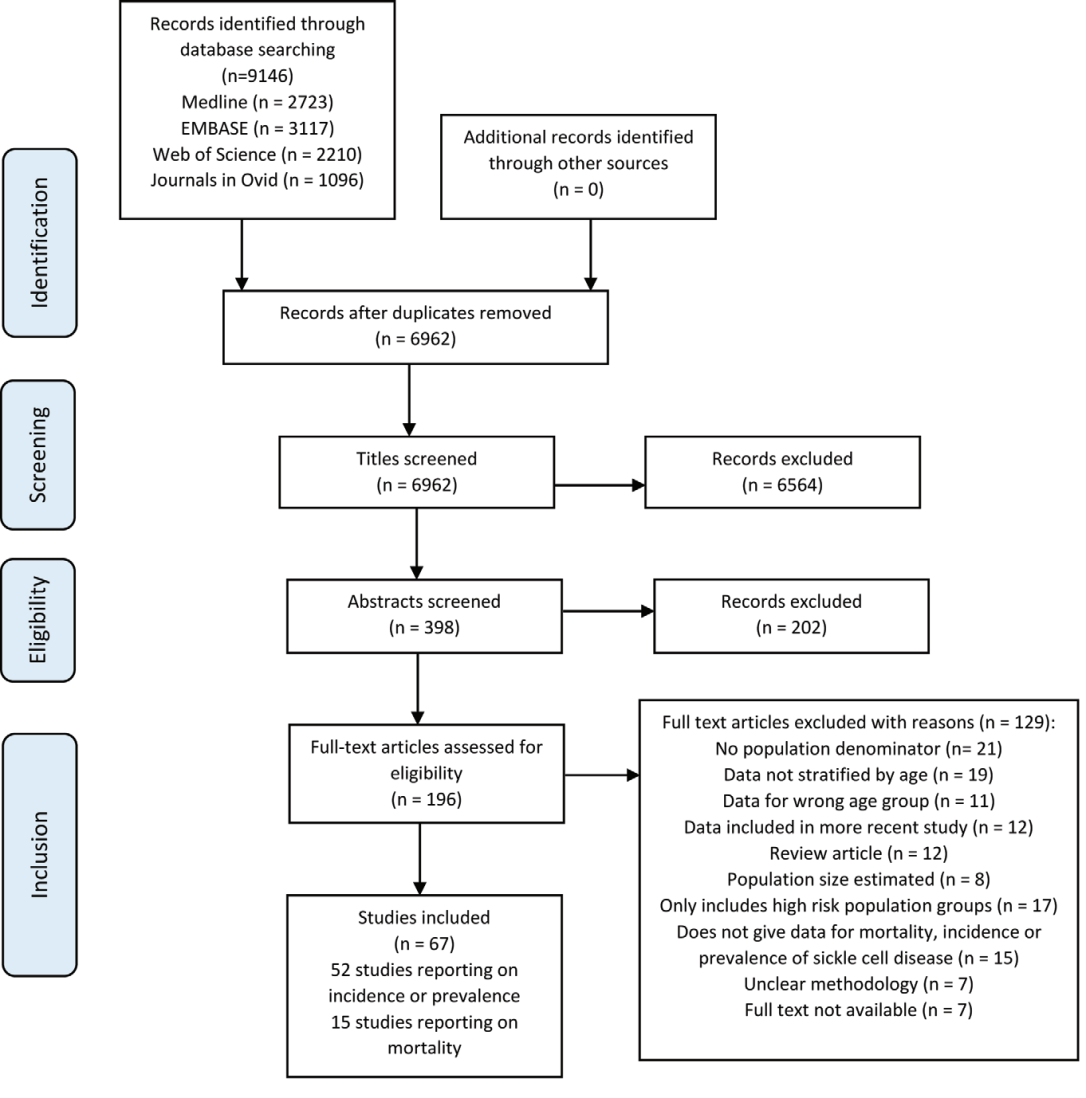
PRISMA flowchart detailing study selection.

The results of the literature search are shown in the flowchart in **Figure 1**. The literature search returned 9146 records, with 67 studies selected (52 on incidence or prevalence, and 15 papers reporting on mortality)

### Study characteristics

From the 52 papers reporting on the incidence or prevalence of SCD, 44 papers reported on incidence and were prospective or retrospective cohort studies of live births. Most studies (n = 22) were from the region of the Americas, followed by 12 from Europe, 7 from Africa, 2 from South East Asia and 1 from the Eastern Mediterranean Region. The duration of the study ranged from 3 months to 26 years with a median duration of 4 years. There were also eight cross-sectional studies reporting on sickle cell disease prevalence in children under five years. Five of these were from Africa, one from the East Mediterranean Region, one from South East Asia and one from the region of the Americas.

There were 15 studies which reported on mortality from sickle cell disease, which were prospective or retrospective cohort studies on groups of children with homozygous sickle cell disease. The sample size was between 52 and 2576, and the duration was from 6 months to 27 years with a median duration of 8 years. There was one from African region, two from Eastern Mediterranean Region, two from European region and 10 from the Americas.

From all 66 studies 42 were rated as high quality and remaining 24 rated as moderate quality. All studies were conducted between 1996 and 2016. Details of all selected studies are in Tables S1, S2 and S3 in **Online Supplementary Document(Online Supplementary Document)**.

### Meta-estimates

**Figure 2:**
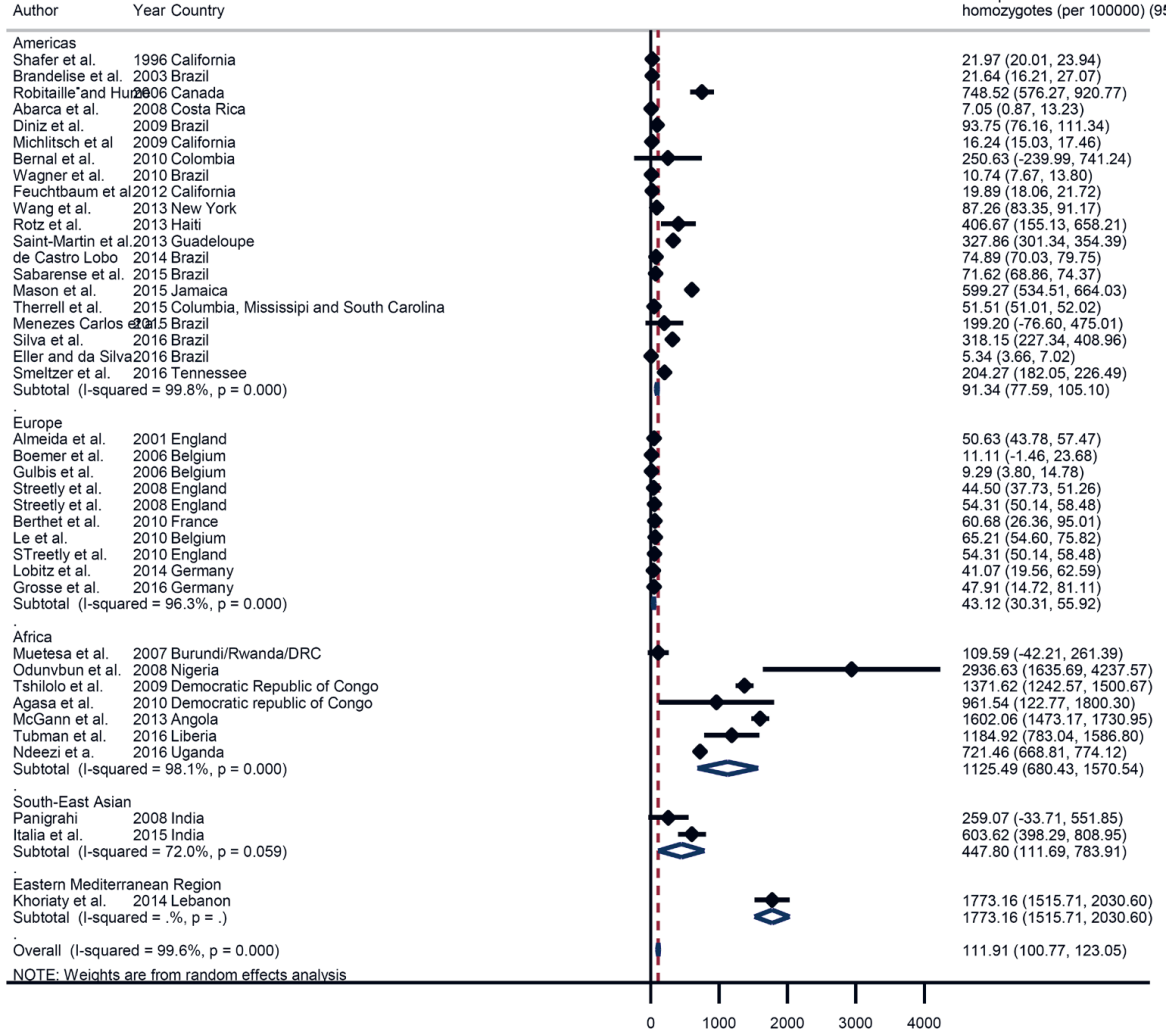
Pooled birth prevalence (per 100 000 live births) of sickle cell disease homozygotes globally and by world regions.

**Figure 3:**
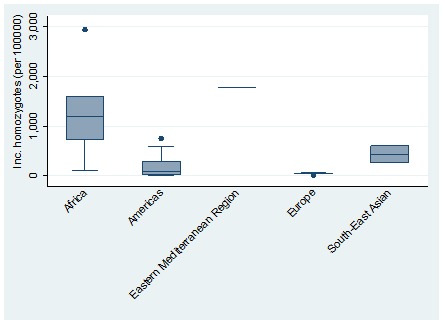
Distribution of birth prevalence (per 100 000 live births) of sickle cell disease homozygotes by world regions. Note: The boxes represent the interquartile range of birth prevalence where the middle 50% (25-75%) of data are distributed; the bars represent birth prevalence outside the middle 50% (<25% or >75%); the dots represent specific birth prevalence which were a lot higher than normally observed (outliers) and the lower, middle and upper horizontal lines represent the minimum, median and maximum birth prevalence (excluding outliers), respectively.

The global meta-estimate for the birth prevalence of homozygous sickle cell disease was 111.91 per 100 000 live births (95% CI = 100.77-123.05) (**Figure 2**). There were however wide disparities by region, with a birth prevalence in Africa of 1125.49 per 100 000 (95% CI = 680.43-1570.54) compared with 43.12 per 100 000 (95% CI = 30.31-55.92) in Europe (**Figure 3**).

**Figure 4:**
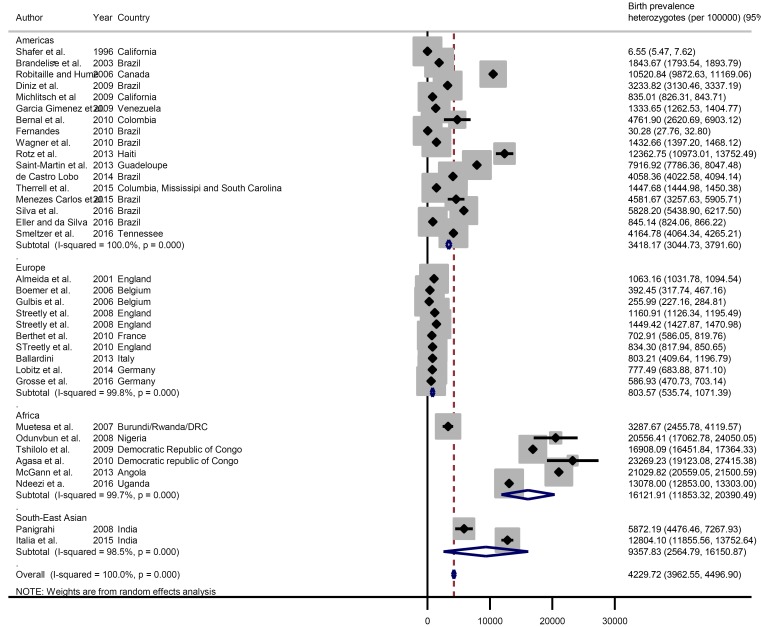
Pooled birth prevalence (per 100 000 live births) of sickle cell disease heterozygotes by world regions.

**Figure 5:**
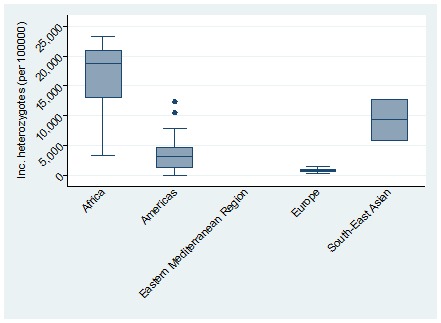
Distribution of birth prevalence (per 100 000 live births) of sickle cell disease heterozygotes by world regions. Note: The boxes represent the interquartile range of birth prevalence where the middle 50% (25-75%) of data are distributed; the bars represent birth prevalence outside the middle 50% (<25% or >75%); the dots represent specific birth prevalence which were a lot higher than normally observed (outliers) and the lower, middle and upper horizontal lines represent the minimum, median and maximum birth prevalence (excluding outliers), respectively.

The global meta-estimate for the birth prevalence of heterozygous sickle cell disease was 4229.72 per 100 000 (95% CI = 3962.55-4496.90) with the highest birth prevalence in Africa: 16 121.91 per 100 000 (95% CI = 11 853.32-20 390.49) and the lowest in Europe 803.57 (95% CI = 535.74-1071.39) (**Figure 4**). There was no data available for the Eastern Mediterranean Region (**Figure 5**).

**Figure 6:**
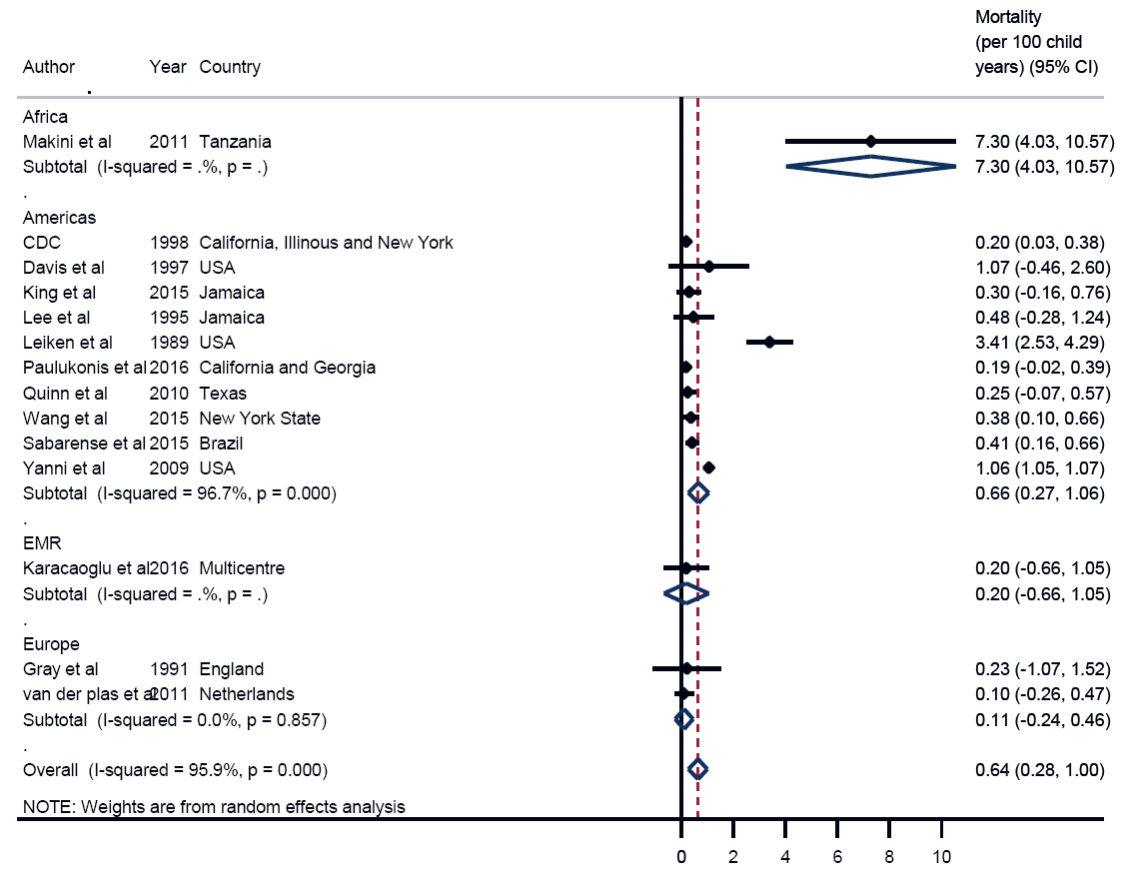
Pooled mortality rate (per 100 child-years of observation) due to sickle cell disease across world regions.

**Figure 7:**
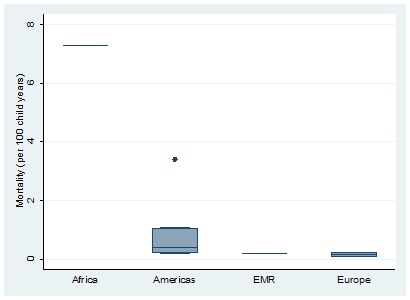
Distribution of sickle-cell disease mortality (per 100 child-years of observation) over world regions. Note: The boxes represent the interquartile range of mortality rate where the middle 50% (25-75%) of data are distributed; the bars represent mortality rate outside the middle 50% (< 25% or > 75%); the dots represent specific mortality rate which were a lot higher than normally observed (outliers) and the lower, middle and upper horizontal lines represent the minimum, median and maximum mortality rate (excluding outliers), respectively.

The overall pooled estimate of mortality from the limited data available was 0.64 per 100 years of child observation (95% CI = 0.28-1.00) (**Figure 6**). Estimates are summarised in the forest plot in Figure 2. There was discrepancy between regions with the lowest rate seen in Europe 0.11 (95% CI = -0.24-0.46) and the highest rate seen in Africa 7.3 (95% CI = 4.03-10.57) (**Figure 7**).

## DISCUSSION

From our pooled meta-estimates, we found the overall mortality rate to be 0.64 per 100 years of child observation with the highest rate in Africa at 7.3 per 100 years of observation. The confidence in these figures however is low due to limited available data. We estimated the global birth prevalence of homozygous SCD to be 0.11%. In 2015, this would amount to about 150 000 births with SCD. This is much lower than the estimates reported by Piel and colleagues [2], with 305800 newborns with sickle cell anemia estimated in 2010. Although the study made several assumptions which could have resulted in their higher estimates, the lack of data on SCD from several African countries, where the prevalence and mortality from SCD are presumed to be higher, could have affected our estimates. For example, in the African Region (with only seven studies), the birth prevalence was ten times higher at 1.1% compared to our overall pooled estimate of 0.11%. Indeed, the higher SCD estimates are expected due to the relationship between sickle cell trait and malaria endemic regions. This presents major concerns as many African nations lack the resources to provide care and follow-up [5]. In addition, malaria precipitates sickling crises and is thought to be the most common cause of childhood mortality in patients with SCD [8].

### Limitations

There were several limitations in data contributing to our estimates. There is a paucity of mortality data available for children under 5 globally, likely a reflection of the fact that in HICs under 5 mortality from SCD is now very low whereas in LMICs many children with SCD die before diagnosis [9]. Only five of the studies included data collected in the last decade, meaning even the data from HIC is likely to be unreflective of the current situation, where SCD is now better understood and there is a greater evidence for treatment options. In LMICs there was only one study in Tanzania reporting on mortality and as this is a single centre study in a sickle cell treatment unit, the generalizability of the results is likely to be limited [10]. Furthermore, much mortality data was poorly characterised and did not provide mortality rate or child years of observation. This meant that in calculating the meta-estimates often the only option was to multiply the sample population by the duration of study to estimate child years of follow up, resulting in mortality figures that are likely under-estimates.

Most incidence and prevalence data were from regions with the lowest incidence of SCD – North America and Europe – in countries where there are reliable birth registries and mandatory neonatal screening. Only six studies from the African region could be included in the meta-estimate for birth prevalence and there was wide variation in estimates from these countries. Because there are few comprehensive national screening programmes in LMICs, the quality of data from these regions was lower, often coming from single centre studies or cross-sectional studies. The lack of national birth registries also meant that much of the data from LMICs, particularly in the African region, only captured births within health centre/hospitals, resulting in selection bias as in Sub-Saharan Africa only half of births occur within health care facilities [11]. Therefore, while we can have reasonable confidence in the meta-estimates of birth prevalence from Europe and America, the data limitations from the other regions mean that our figures are likely to be under-estimates of the disease burden.

### Findings in context

It is clear from our results that SCD is an important contributor to under 5 mortality in LMICs, particularly sub-Saharan Africa. The wide discrepancy between mortality in these settings compared with HIC suggests that much of this mortality is largely preventable. There is considerable evidence for comprehensive SCD care programmes as a way to improve outcomes for children with SCD, providing penicillin and malarial chemoprophylaxis as well as family education [5,12,13]. There has been some success piloting such programmes in LMICs, for example a trial of holistic family education in Benin showed a significant improvement in general health of children with SCD and a marked reduction in acute SCD events [12]. A similar program in Nigeria showed a reduction in mortality from 20.6% to 0.6%, as well as markedly reduced hospital admissions and transfusion requirements [13]. Due to the added risk of malaria in tropical regions, any such programmes must be combined with adequate malaria treatment strategies and all children with SCD should be on lifelong malarial chemoprophylaxis [8]. Hopefully lessons learned from these programmes can be used to increase the coverage of these much-needed interventions [1], however the difficulty is in securing sustainable funding to continue such programmes, particularly in a current political climate where NCDs are under-recognised and under-funded by the international community [5].

Enhanced diagnostics to enable early case-identification will also be essential in improving SCD outcomes. Currently neonatal screening is not widely available in many LMICs and many children die having never been diagnosed [14]. One important step in tackling SCD, therefore, could be the introduction of national comprehensive newborn screening programmes. Laboratory and staffing constraints make this a challenging task to accomplish, particularly as currently the diagnosis of SCD relies on relatively sophisticated techniques of high performance liquid chromatography or iso-electric focussing [1]. There has been some progress in the development of a rapid diagnostic test for SCD and if this could be widely and cheaply available, it could be powerful in early detection of cases [6,15].

## CONCLUSION

SCD is an under-recognised and under-funded cause of under 5 mortality, particularly in Africa. There are several well recognised and well evidenced ways to improve outcomes and the development of integrated treatment pathways for SCD sufferers would be a powerful way to tackle this excess mortality and morbidity. Comprehensive newborn screening programmes are a crucial part of attaining this and this may become more achievable with the promise of a rapid-diagnostic test on the horizon. The current evidence base is limited and there is a need for large scale prospective cohort studies to accurately assess the SCD burden. For any headway to be made in tackling SCD, funding needs to be reprioritised to bring NCDs to the fore, particularly genetic disease such as this one which have received little global investment to date.
