# Supplementary material for: The global burden of sickle cell disease in children under five years of age: a systematic review and meta-analysis
Source: J Glob Health. 2018 Dec 7;8(2):021103. doi: 10.7189/jogh.08.021103 (PMC6286674; doi:10.7189/jogh.08.021103)
Supplement: Online Supplementary Document [file jogh-08-021103-s001.pdf]

Online Supplementary Document

Wastnedge et al. The global burden of sickle cell disease in children under five years of age: a systematic review and meta-analysis

J Glob Health 2018;8:021103

Table S1. Birth prevalence estimates of homozygous and heterozygous SCD from all studies

Region	Authors	Year	Country	Study duration	Sample size	No. homozy-gotes	Birth prevalence	Number heterozygotes	Birth prevalence
**AFR**	Muetesa et al.	2007	Burundi/Rwanda/DRC	2.0	1825	2	109.6	60.0	3287.7
	Odunvbun et al.	2008	Nigeria	0.3	647	19	2936.6	133.0	20556.4
	Tshilolo et al.	2009	Democratic Republic of Congo		31204	428	1371.6	5276.0	16908.1
	Agasa et al.	2010	Democratic republic of Congo	0.3	520	5	961.5	121.0	23269.2
	McGann et al.	2013	Angola	2.0	36453	584	1602.1	7666.0	21029.8
	Tubman et al.	2016	Liberia	1.1	2785	33	1184.9		
	Ndeezi et a.	2016	Uganda	1.0	99243	716	721.5	12979.0	13078.0
**AMR**	Shafer et al.	1996	California	4.0	2184378	480	22.0	143.0	6.5
	Brandelise et al.	2003	Brazil	18.0	281884	61	21.6	5197.0	1843.7
	Robitaille and Hume	2006	Canada	15.0	9619	72	748.5	1012.0	10520.8
	Abarca et al.	2008	Costa Rica	1.0	70943	5	7.0		
	Diniz et al.	2009	Brazil	3.0	116271	109	93.7	3760.0	3233.8
	Michlitsch et al	2009	California	8.5	4236000	688	16.2	35371.0	835.0
	Garcia Gimenez et al.	2009	Venezuela		101301		0.0	1351.0	1333.6
	Bernal et al.	2010	Colombia	1.0	399	1	250.6	19.0	4761.9
	Fernandes	2010	Brazil	7.0	1833030		0.0	555.0	30.3
	Wagner et al.	2010	Brazil		437787	47	10.7	6272.0	1432.7
	Feuchtbaum et al.	2012	California	5.0	2282138	454	19.9		
	Wang et al.	2013	New York	8.0	2189967	1911	87.3		
	Rotz et al.	2013	Haiti	1.0	2459	10	406.7	304.0	12362.7
	Saint-Martin et al.	2013	Guadeloupe	26.0	178428	585	327.9	14126.0	7916.9

	de Castro Lobo	2014	Brazil	10.0	1217833	912	74.9	49424.0	4058.4
	Sabarense et al.	2015	Brazil	14.0	3617919	2591	71.6		
	Mason et al.	2015	Jamaica	7.0	54566	327	599.3		
	Therrell et al.	2015	Columbia, Mississipi and South Carolina	20.0	76527627	39422	51.5	1107875.0	1447.7
	Menezes Carlos et al.	2015	Brazil	2.3	1004	2	199.2	46.0	4581.7
	Silva et al.	2016	Brazil	4.0	14773	47	318.1	861.0	5828.2
	Eller and da Silva	2016	Brazil	10.0	730412	39	5.3	6173.0	845.1
	Smeltzer et al.	2016	Tennessee	10.0	158616	324	204.3	6606.0	4164.8
**EMR**	Khoriaty et al.	2014	Lebanon	2.3	10095	179	1773.2		
**EUR**	Almeida et al.	2001	England	10.0	414801	210	50.6	4410.0	1063.2
	Boemer et al.	2006	Belgium	3.0	27010	3	11.1	106.0	392.4
	Gulbis et al.	2006	Belgium	10.0	118366	11	9.3	303.0	256.0
	Streetly et al.	2008	England	2.0	373069	166	44.5	4331.0	1160.9
	Streetly et al.	2008	England	2.0	1198614	651	54.3	17373.0	1449.4
	Lopez-Escribano	2009	Spain						
	Berthet et al.	2010	France	9.0	19775	12	60.7	139.0	702.9
	Le et al.	2010	Belgium	16.0	222352	145	65.2		
	Streetly et al.	2010	England	2.0	1198614	651	54.3	10000.0	834.3
	Ballardini et al.	2013	Italy	1.3	1992		0.0	16.0	803.2
	Lobitz et al.	2014	Germany	1.2	34084	14	41.1	265.0	777.5
	Grosse et al.	2016	Germany	1.2	16697	8	47.9	98.0	586.9
**SEAR**	Panigrahi et al.	2008	India	1.0	1158	3	259.1	68.0	5872.2
	Italia et al.	2015	India	2.0	5467	33	603.6	700.0	12804.1

Table S2. Cross-sectional studies showing prevalence of sickle cell disease

Region	Authors	Year	Country	Study duration	Percentage homozygotes	Percentage heterozygotes	Age range
**AFR**	McGann et al.	2016	Malawi	5 months	0.09	9.94	6-59months
	Simbauranga et al.	2015	Tanzania	4 months	0.00	18.97	6-59months
	Suchdev et al.	2014	Kenya	1 month	1.59	0.17	6-35 months
	Okwi et al.	2010	Uganda		1.28	0.00	6-59months
	Ughasoro et al.	2007	Nigeria	1 year	1.72	0.00	6-59months
**AMR**	Cardoso et al.	2012	Brazil	1 month	0.19	8.94	6-59months
**EMR**	Daak et al.	2016	Sudan	1 month	1.92	17.31	0-59 months

**SEAR**	Panigrahi et al.	2015	India	2 years	2.29	12.70	0-59 months

Table S3. Studies reporting on mortality from sickle cell disease

Region	Authors	Year	Country	Study duration	Sample size	Number of deaths	Mortality rate (per 100 child years observation)	Age range
**AFR**	Makini et al	2011	Tanzania	5	243	22	7.30	0-59 months
**AMR**	CDC	1998	California, Illinous and New York	4	2487	20	0.20	0-59 months
	Davis et al	1997	USA	24	173.2866044	1068	1.07	12-59 months
	Hamideh and Alvarez	2013	USA	10				0-59 months
	King et al	2015	Jamaica	14	548	8	0.30	0-59 months
	Lee et al	1995	Jamaica	8	315	12	0.48	0-59 months
	Leiken et al	1989	USA	0.5	1643	28	3.41	0-36 months
	Paulukonis et al	2016	California and Georgia	4	1738	13	0.19	0-59 months
	Quinn et al	2010	Texas	27	940	14	0.25	0-59 months
	Wang et al	2015	New York State	8	1911	27	0.38	0-24 months
	Sabarense et al	2015	Brazil	14	2576	148	0.41	0-59 months
	Yanni et al	2009	USA	7	2649166	197	1.06	0-36 months
**EMR**	Karacaoglu et al	2016	Multicentre	5	102	1	0.20	0-59 months
**EUR**	Gray et al	1991	England	17	52	2	0.23	0-59 months
	van der plas et al	2011	Netherlands	22	298	4	0.10	0-59 months
